# Risk Factors for *Mycobacterium ulcerans* Infection, Southeastern Australia

**DOI:** 10.3201/eid1311.061206

**Published:** 2007-11

**Authors:** Tricia Y.J. Quek, Eugene Athan, Margaret J. Henry, Julie A. Pasco, Jane Redden-Hoare, Andrew Hughes, Paul D.R. Johnson

**Affiliations:** * University of Melbourne, Melbourne, Victoria, Australia; † Barwon Health, Geelong, Victoria, Australia; ‡ Barwon Health Centre of Education & Practice Development, Geelong, Victoria, Australia; § Austin Health, Melbourne, Victoria, Australia

**Keywords:** Mycobacterium ulcerans, Mycobacterium infections, atypical, communicable diseases, lifestyle, insect vectors, case-control studies, Australia, epidemiology, research

## Abstract

Buruli/Bairnsdale ulcer (BU) is a severe skin and soft tissue disease caused by *Mycobacterium ulcerans.* To better understand how BU is acquired, we conducted a case–control study during a sustained outbreak in temperate southeastern Australia. We recruited 49 adult patients with BU and 609 control participants from a newly recognized BU-endemic area in southeastern Australia. Participants were asked about their lifestyle and insect exposure. Odds ratios were calculated by using logistic regression and were adjusted for age and location of residence. Odds of having BU were at least halved for those who frequently used insect repellent, wore long trousers outdoors, and immediately washed minor skin wounds; odds were at least doubled for those who received mosquito bites on the lower legs or lower arms. This study provides new circumstantial evidence that implicates mosquitoes in the transmission of *M. ulcerans* in southeastern Australia.

## Abstract 2

Epidemiologic evidence shows that mosquitoes play a role in transmission to humans.

*Mycobacterium ulcerans* is an environmental pathogen that is most commonly associated with water and soil (*1*–*4*). In humans, *M. ulcerans* causes an ulcerative skin disease known as Buruli ulcer in Africa and Bairnsdale ulcer in southeastern Australia (BU). Although BU is usually regarded as a disease of subtropical climates, a slowly increasing number of cases have been recorded in temperate southeastern Australia over the past 15 years. In sub-Saharan Africa countries such as Côte d’Ivoire (*3*), Uganda (*5*), Benin (*6*), and Ghana (*7*), BU has been responsible for considerable suffering and disability, and the disease has become more common than tuberculosis and leprosy in some highly disease-endemic regions (*8*).

*M. ulcerans* infection was first definitively described in 1948 (*9*). In the state of Victoria, in southeastern Australia, BU cases appeared to be confined to the original BU-endemic region of Bairnsdale until 1982, when a new disease focus was noted 150–200 km west of Bairnsdale (Figure). Since 1991, incidence has progressively risen, and outbreaks of BU have occurred at Phillip Island and on the Mornington Peninsula (*10*). In 1998, BU was reported >50 km farther west on the Bellarine Peninsula, and since then, a sustained outbreak has involved at least 3 Bellarine Peninsula towns. The annual number of cases in Victoria has continued to increase; 41 cases were reported to the Victorian Department of Human Services in 2005, compared with 25 in 2004 and 12 in 2003. Although the incidence is still low overall, the disease is becoming a substantial public health concern in affected coastal communities. Treatment of BU is not straightforward and usually requires surgery combined with prolonged courses of antimicrobial drugs. The average cost of diagnosing and treating a case of BU in Australia in 2004 was estimated to be AUD $14,608 (*11*).

**Figure:**
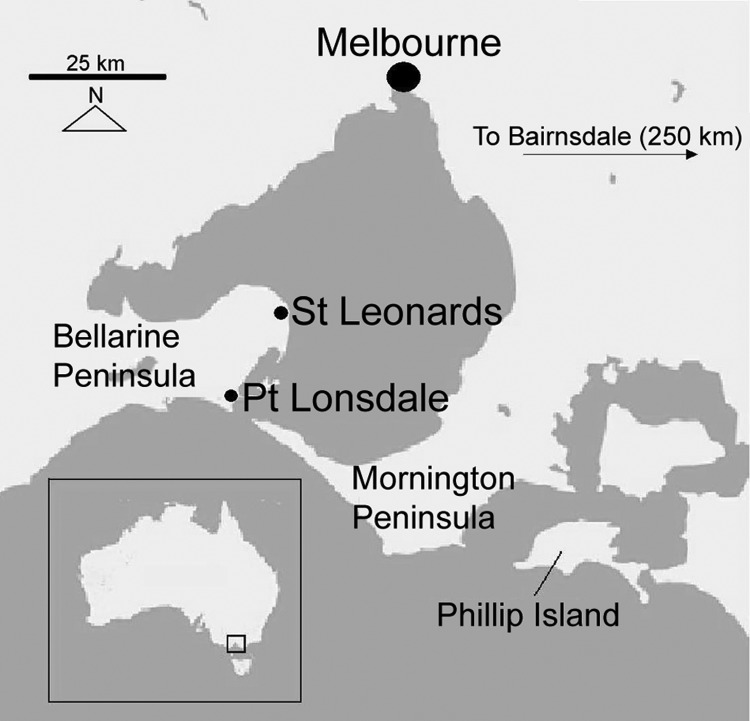
Bellarine Peninsula region, southeastern Australia.

Although the mode of *M. ulcerans* transmission is unknown, epidemiologic evidence suggests that transmission occurs through being in close proximity to slow-moving waters contaminated with the bacteria (*1*–*3*,*12*,*13*). Previous case–control studies have reported an increased risk for BU for those who regularly swim or wade through rivers (*12*,*13*) and those who farm near a BU-endemic town’s main rivers (*3*). More recently, results from laboratory experiments have suggested a new hypothesis that aquatic insects, fish, and plants may be reservoirs for *M. ulcerans* (*14*–*17*) and that aquatic insects may even be involved in transmission to humans (*14*,*16*,*18*).

Because the ecologic factors that are driving the emergence of *M. ulcerans* infection in newly disease-endemic countries are unknown, the most effective means of reducing the incidence of BU may be to identify modifiable behavior associated with the risk for infection. Therefore, we designed a case–control study to investigate risk factors that could be used to assist public health authorities in reducing the effects of BU.

## Methods

### Study Area

This study was conducted on the Bellarine Peninsula, in southeastern Australia, on which *M. ulcerans* infection is newly endemic (Figure). Most residents live in small towns along the coast of the Peninsula (e.g., Point Lonsdale, St Leonards, Queenscliff, Barwon Heads), while the more sparsely populated interior is used for primary agricultural production and industry.

### Participants

BU has been a reportable disease in Victoria since 2004. Patients with BU were identified retrospectively from the records of the treating physicians at Barwon Health and Austin Health from 1998 through 2005 and from the Victorian Department of Human Services reportable diseases database. The preliminary search found 73 confirmed BU case-patients, defined as patients from whom a clinical specimen (swab or biopsy) was positive for *M. ulcerans* by culture, PCR, or both (*19*). All patients in this study were either permanent residents of the Bellarine Peninsula or persons who had visited the area before first noticing the lesion that was subsequently diagnosed as BU. Patients were excluded if they were <20 years of age (n = 9), had moved with no forwarding address (n = 2), died (n = 1), or had a diagnosis of dementia (n = 1). Of the remaining 60 case-patients who were contacted, 49 responded (82% participation rate) and median age was 70 years (interquartile range 58–81 years).

A total of 609 controls participated in the study (78% participation rate). Control participants were randomly selected from the Commonwealth electoral roll (Australian Electoral Commission, 2005) for Point Lonsdale and St Leonards (which have each experienced separate outbreaks of BU since 1998) and other towns on the Bellarine Peninsula (e.g., Clifton Springs, Curlewis, Whittington, Portarlington, Queenscliff, Ocean Grove, Indented Head, and Drysdale). The entire adult population of the Bellarine Peninsula is captured by the Australian electoral roll because it is compulsory for all Australian citizens to vote in federal elections. We were therefore able to randomly select controls from the entire adult population of 32,480 on the Bellarine Peninsula (*20*).

### Data Collection

Case-patients and control participants were mailed a written questionnaire with questions about their medical history; outdoor lifestyle and behavior; and soil, animal, and insect exposure on the Bellarine Peninsula. Case-patients were asked to restrict their responses to the 12-month period before their diagnosis of BU. We investigated outdoor-related behavior and habits such as types of clothing worn, measures taken to protect against insect bites, types of treatment given to skin trauma, parts of their bodies most frequently bitten by insects, natural fauna and household pets with which they had regular contact, and types of soil products they were exposed to in the previous year while on the Peninsula. For activities associated with water or located near specified lakes or marshes on the Bellarine Peninsula, and for exposure to mosquitoes (Family Culicidae), March flies (Family Tabanidae), and sandflies (Family Ceratopogonidae), the responses were subdivided according to season (summer, autumn, winter, and spring).

### Statistical Analyses

Analyses were performed by using Minitab statistical software, version 14 (Minitab Inc., State College, PA, USA). The ages of the case-patients and control participants were compared by using a 2-sample *t* test. Chi-square analysis, Fisher exact test, and logistic regression determined the odds ratio (OR) and 95% confidence interval (CI) for BU after adjusting for age and town of residence (Point Lonsdale, St Leonards, or all other towns on the Bellarine Peninsula). Multivariate models were determined by applying a backward elimination technique to the logistic regression while adjusting for age and town of residence. In all statistical models, p values <0.05 were considered statistically significant.

### Ethics

The Barwon Health Research and Ethics Advisory Committee and the Victorian Department of Human Services approved the study. Consent to participate in the study was implied by those who returned the self-completed written questionnaire.

## Results

Characteristics of the case-patients and control participants are shown in Table 1. Both sexes were equally affected by BU. Most case-patients were elderly adults, and their median age was higher than that of the control participants (p = 0.01). Among case-patients, ≈69% reported that they were either permanent residents of Point Lonsdale or had visited the town ≈3 months before onset of symptoms. Personal health factors such as cancer, immunosuppressive medication taken within the previous year, or having had an *M. bovis* BCG vaccination did not alter the odds of having BU.

**Table 1:** Characteristics of 49 Buruli ulcer case-patients and 609 control-participants, southeastern Australia*

Variable	Case-patients, n (%)	Control-participants, n (%)	p value
Female sex	25 (51)	326 (54)	0.90
Towns in which most time on Bellarine Peninsula was spent			<0.01
Point Lonsdale	34 (69)	212 (35)	
St Leonards	8 (16)	202 (33)	
Other	7 (14)	195 (32)	
Health condition			
Diabetes	1 (2)	38 (6)	0.23
Cancer	5 (10)	40 (7)	0.57
Immunosuppressive medication	2 (4)	36 (6)	0.45
*Mycobacterium bovis* BCG immunization	16 (33)	174 (29)	0.30
Regular tobacco smoker	3 (6)	71 (12)	0.37

*Median age (interquartile range) for case-patients was 70 (58–82) years and for control participants 61 (48–72) years; p = 0.01.

Table 2 displays the participants’ behavioral and lifestyle choices on the Bellarine Peninsula. Wearing insect repellent (OR 0.38, 95% CI 0.20–0.71) or wearing long trousers when outdoors (OR 0.51, 95% CI 0.27–0.97) were each found to reduce the odds of having BU. Immediately washing a wound sustained outdoors was also found to decrease the odds for disease (OR 0.47, 95% CI 0.24–0.94). Owning a household pet or using gardening products such as fertilizer, potting mix, and topsoil was not found to be associated with BU. Participants were also assessed on whether they frequented any of 8 prominent lakes or marshes in Point Lonsdale or St Leonards. Case-patients were more likely than control participants to have visited a small ornamental lake at the western edge of Point Lonsdale during autumn (p = 0.04), but associations with all the other water areas surveyed were not statistically significant.

**Table 2:** Association of Buruli ulcer with behavioral and lifestyle factors, Bellarine Peninsula, southeastern Australia

Variable	Case-patients, n (%)	Control-participants, n (%)	OR (95% CI)*	p value
Outdoor clothing covers				
Arms	18 (38)	247 (41)	0.62 (0.33–1.18)	0.15
Legs	24 (50)	343 (57)	**0.51 (0.27–0.97)**	**0.04**
Immediately washes wounds	12 (24)	225 (37)	**0.47 (0.24–0.94)**	**0.03**
Uses gardening products				
Potting mix	32 (67)	369 (61)	1.28 (0.68–2.40)	0.44
Pesticides	20 (42)	187 (31)	1.51 (0.83–2.77)	0.18
Fertilizers	30 (63)	307 (50)	1.28 (0.68–2.40)	0.44
Had topsoil delivered to home	6 (13)	65 (11)	1.34 (0.54–3.31)	0.53
Construction site is				
At home	5 (10)	64 (11)	1.24 (0.47–3.32)	0.66
Near home	6 (13)	155 (25)	0.48 (0.20–1.17)	0.11
Wears gardening gloves				
Does not garden	2 (4)	64 (11)	0.36 (0.08–1.56)	0.17
Always/usually	20 (42)	223 (37)	0.97 (0.52–1.80)	0.92
Sometimes/never†	26 (54)	321 (53)		
Washes hands after gardening				
Does not garden	2 (4)	65 (11)	0.33 (0.08–1.42)	0.07
Always/usually	43 (90)	515 (85)	0.61 (0.23–1.61)	0.24
Sometimes/never†	3 (6)	24 (4)		
Owns a pet	21 (43)	343 (56)	0.74 (0.39–1.40)	0.35
Wears insect repellent	15 (31)	328 (54)	0.38 (0.20–0.71)	**<0.01**
Ever visits water area on western edge of Point Lonsdale				
Summer	9 (19)	57 (9)	–	0.08‡
Autumn	9 (19)	49 (8)	–	**0.04‡**
Winter	8 (17)	46 (8)	–	0.07‡
Spring	7 (15)	50 (8)	–	0.22‡

*OR, odds ratio; CI, confidence interval. OR (95% CI) adjusted for age and location; **boldface** indicates statistical significance; – indicates that ORs could not be calculated. †Denotes reference group. ‡Fisher exact test for small numbers.

The extent of insect exposure on the Bellarine Peninsula is shown in Table 3. The odds more than doubled if participants reported that they had received mosquito bites on commonly exposed sites of the body, i.e., lower arms (OR 2.56, 95% CI 1.23–5.33) and legs (OR 2.51, 95% CI 1.18–5.31). The same biting pattern was not noted in response to the same questions regarding bites from March flies and sandflies.

**Table 3:** Insect exposure as risk factors for Buruli ulcer on the Bellarine Peninsula, southeastern Australia

Variable	Case-patients, n (%)	Control-participants, n (%)	OR (95% CI)*	p value
Season when frequently bitten by mosquitoes				
Summer	24 (49)	373 (62)	0.71 (0.39–1.30)	0.27
Autumn	16 (33)	238 (39)	0.94 (0.49–1.79)	0.85
Winter	7 (15)	37 (6)	–	0.07†
Spring	12 (25)	189 (31)	0.88 (0.44–1.75)	0.71
Area most often bitten by mosquitoes				
Head	18 (37)	290 (48)	0.61 (0.33–1.12)	0.11
Upper arms	30 (61)	353 (58)	1.37 (0.74–2.55)	0.31
Forearms	39 (80)	422 (69)	**2.56** (**1.23–5.33**)	**0.01**
Hands	28 (57)	381 (63)	1.36 (0.71–2.62)	0.36
Torso	5 (10)	128 (21)	0.48 (0.18–1.23)	0.13
Upper legs	8 (16)	80 (13)	1.67 (0.73–3.82)	0.23
Lower legs	39 (80)	423 (69)	**2.51** (**1.18–5.31**)	**0.02**
Feet	11 (22)	175 (29)	0.94 (0.45–1.97)	0.88

*OR, odds ratio; CI, confidence interval. OR (95% CI) adjusted for age and location; **boldface** indicates statistical significance; – indicates that ORs could not be calculated. †Fisher exact test for small numbers.

A variety of outdoor activities were surveyed, including beach activities, freshwater or salt water swimming and fishing, surfing, sailing, bushwalking, lawn bowling, golf, bird watching, cycling, and gardening. None of these activities was associated with BU.

A backward elimination technique was applied to further investigate all significant factors. In a multivariate model, after adjusting for age and location, insect repellent use (OR 0.37, 95% CI 0.19–0.69) and being bitten by mosquitoes on the lower legs (OR 2.60, 95% CI 1.22–5.53) were found to be independently associated with BU.

## Discussion

BU occurs in focal outbreaks in >30 countries worldwide. Although the disease has been long neglected (*21*), substantial progress has been recently made and includes the development of rapid diagnosis by PCR (*19*), the discovery of the lipid toxin that explains the destructive nature of the disease (*22*), and the identification of the virulence plasmid that harbors the genes that produce the toxin (*23*). Also, increasing evidence supports the use of drug therapy for BU (*24**–**26*), which until recently has been regarded as a disease that would respond only to surgery. However, the ecologic factors driving the increasing incidence of BU in different regions of the world and the mode of transmission to humans who live in BU-endemic areas remain to be determined.

One of the major differences between BU in southeastern Australia and BU in sub-Saharan Africa is the proportion of older persons affected. Of the total number of cases linked to the Bellarine Peninsula, 33 (67%) case-patients were >60 years of age, whereas many studies from Africa report that most BU cases occur in children <15 years of age (*3*,*6*,*7*,*27*). A high proportion of the permanent residents of the Bellarine Peninsula are older Australians who have relocated to the coast for their retirement. However, especially during summer, young families visit the area and increase the local population considerably. Although at least 9 children or teenagers have acquired BU linked to the Bellarine Peninsula, advanced age may be an independent risk factor for BU, as was reported recently in Benin (*28*,*29*).

Our results suggest that protecting the body from environmental exposure appears to reduce the odds for disease. These measures include wearing long trousers and immediately washing wounds after sustaining minor skin trauma; such measures have also been identified as protective factors against BU in west Africa (*3*,*13*,*30*). These findings support the established hypothesis of *M. ulcerans* transmission, which postulates that direct contact with the skin is required for transmission to occur. Negative findings with regard to animal exposure factors (domestic pets and wildlife) also make it unlikely that contact with these animals is a risk factor for infection with *M. ulcerans* on the Bellarine Peninsula, although the bacterium has been isolated from skin ulcers in several wild animals near the original Bairnsdale region and other BU-endemic areas in Victoria (*31**,**32*).

Only 1 case-patient in this study reported having had direct physical contact with environmental water (other than the ocean) from a BU-endemic town, and only 14 (2%) of control participants reported having swum in fresh water on the Bellarine Peninsula (data not shown). Case-patients were more likely than control participants to have visited only 1 of our surveyed water areas, a small park reserve (Table 2), although the low numbers of positive responses to this question limited our ability to draw further insight from these data. A small percentage of participants reported having spent time near marshy areas on the Peninsula, which suggests that transmission is most likely to occur indirectly or that *M. ulcerans* has spread to areas outside of its presumed aquatic environment. Previous studies conducted in BU-endemic African countries found that case-patients were more likely to report that they regularly immersed themselves in, or worked along, rivers or marshy areas (*12*). On the Bellarine Peninsula, contact with lakes and rivers is not a large part of the lifestyle because recreational swimming and boating activities are largely based around coastal saltwater areas. This difference in lifestyle could also possibly explain the lower incidence rate of BU in Australia than in the highly affected African regions.

More case-patients than control-participants reported having been bitten by mosquitoes on the distal areas of their arms and legs, the sites most commonly exposed to the environment. Although the responses to our insect-exposure questions suggest an association between mosquito bites and *M. ulcerans*, this association may be just a nonspecific marker of environmental exposure. However, use of insect repellent remained an independent predictor of reduced risk, which suggests that insects themselves may play a role in transmission. Although no case-control study can establish causation, our findings are consistent with the hypothesis that mosquitoes play a role in transmission of *M. ulcerans* to humans, at least in southeastern Australia. In a previous outbreak of BU in Australia in the 1990s, contaminated aerosolized water droplets that could be inhaled or ingested were suggested as a means of infection for persons who did not have direct contact with the likely point source. Although mosquitoes and other insects were not tested during that outbreak, they also could have played a role in transmission (*1*,*33*).

We acknowledge several limitations in our study. Reliability of patient recall may be biased because some BU cases date back to 1998. However, the median time between the date of completing the questionnaire and the time of diagnosis of BU was 1.51 years (interquartile range 0.90–3.29 years). Avoidance of recall bias is impossible because the incubation period varies by several months, and delay between first noticing a skin lesion and the diagnosis of BU is often substantial (*34*,*35*). Studies with more cases would help validate and refine the protective and risk factors identified in this study.

Despite many differences between the BU-endemic areas in southeastern Australia and western Africa, case-control studies in these diverse environments produce surprisingly concordant results. Direct contact with the environment appears to be a universal risk factor for acquisition of BU, and use of protective clothing appears to reduce this risk. In this study we also established a new risk factor—exposure to mosquitoes. Our data support an association between mosquitoes and *M. ulcerans* on the Bellarine Peninsula during the outbreak and demonstrate that BU case-patients were likely to have been bitten by mosquitoes. We hypothesize that in this BU-endemic region, mosquitoes become colonized or passively contaminated when they come into contact with *M. ulcerans* in the environment and then transmit it to humans living nearby. However, before introducing specific public health interventions, such as mosquito control and campaigns to encourage the use of repellents and protective clothing for those who live in BU-endemic areas, the presence of *M. ulcerans* in mosquitoes (*36**–**38*) from the BU-endemic area should be confirmed and transmissibility under laboratory conditions should be demonstrated.
